# High Bendability of Short RNA-DNA Hybrid Duplex Revealed by Single-Molecule Cyclization and Molecular Dynamics Simulations

**DOI:** 10.3390/biom15050724

**Published:** 2025-05-15

**Authors:** Bin Wu, Fujia Tian, Yajun Yang, Liang Dai, Xinghua Zhang

**Affiliations:** 1 Hubei Key Laboratory of Cell Homeostasis, College of Life Sciences, Wuhan University, Wuhan 430072, China; binwuu@whu.edu.cn (B.W.); yangyajun@whu.edu.cn (Y.Y.); 2 Department of Physics, City University of Hong Kong, Hong Kong, China; liangdai@cityu.edu.hk

**Keywords:** RNA-DNA hybrid duplex, R-loop, bendability, single-molecule cyclization, molecular dynamics simulations

## Abstract

R-loops are nucleic acid structures composed of an RNA-DNA hybrid (RDH) duplex and a displaced single-stranded DNA (ssDNA), which are fundamentally involved in key biological functions, including transcription and the preservation of genome stability. In an R-loop, the RDH duplex is bent by the folded secondary structures of the displaced ssDNA. Previous experiments and simulations indicated the high bendability of DNA below the persistence length. However, the bendability of a short RDH duplex remains unclear. Here, we report that an RDH duplex exhibits higher bendability than a DNA duplex on the short length scale using single-molecule cyclization experiments. Our molecular dynamics simulations show that an RDH duplex has larger intrinsic curvature and structural fluctuations and more easily forms kinks than DNA, which promote the bending flexibility of RDH from unlooped structures. Interestingly, we found that an RDH duplex composed of a C-rich DNA strand and a G-rich RNA strand shows significantly higher bendability than that composed of a G-rich DNA strand and a C-rich RNA strand in the same CpG island promoter regions, which may contribute to the formation of an R-loop. These findings shape our understanding towards biological processes involving R-loops through the high and sequence-dependent bendability of an RDH duplex.

## 1. Introduction

An R-loop is formed when single-stranded RNA (ssRNA) anneals to its double-stranded DNA (dsDNA) template during or after transcription, generating an RNA–DNA hybrid (RDH) duplex and a displaced single-stranded DNA (ssDNA) [1]. R-loops can occur in the course of transcription [2] or at telomeric regions [3], serving as initiators of genomic instability [2,3], modulators of gene expression [4], and regulators of chromatin-associated epigenetic modifications [5]. R-loops show a strong association with unmethylated CpG island (CGI) promoters and are characterized by pronounced strand-specific GC skew in guanine and cytosine distribution [6,7]. Moreover, the co-transcriptional formation of an R-loop protects CGI promoters from DNA methylation [6].

RDH duplexes account for up to 5% of the mammalian genome [8] and around 8% of the budding yeast genome [9]. RDH-forming sequences are found in approximately 59% of human genes [10]. RDH duplexes play important roles in many biological processes, including transcription, replication, chromosome segregation, telomere regulation, and DNA repair [1,3,11,12,13,14,15,16,17,18]. In general, human cells have developed different mechanisms to resolve RDH duplexes [19]. But there are risks of genomic instability and cell proliferation with abnormally high levels or the wrong context [1,13,20], potentially resulting in human diseases such as cancers and neurological disorders [21,22,23,24,25]. RDH duplexes are also involved in numerous applications in biotechnology, such as fluorescence in situ hybridization (FISH) [26], CRISPR-based genome editing [27,28], nanotechnology [29], and disease treatment [30].

The biophysical properties of an RDH duplex have been explored from multiple perspectives. The RDH duplex adopts an intermediate conformation between B-form DNA and A-form RNA, though it more closely resembles the structural characteristics of A-form RNA [31,32,33,34,35,36,37]. The thermodynamic stability of an RDH duplex highly depends on the sequence [35,38,39,40], with a higher GC content or an increased proportion of pyrimidines in the DNA strand (dPy) contributing to enhanced RDH duplex stability [40,41]. In general, the biological roles of RDH duplexes are closely linked to their mechanical properties. Thus, mechanical properties including the stretching modulus, conformational transitions, and twist stiffness of the RDH duplex have been studied extensively [42,43,44]. Additionally, RDH duplexes are frequently employed as handles in many single-molecule investigations studying RNA structures, including riboswitches, hairpins, and pseudoknots [45,46,47,48].

As one of the mechanical properties, the bending of an RDH duplex is crucial in protein–nucleic acid recognition [32]. The local bending of an RDH duplex is required for the formation of a CRISPR-Cas9 R-loop complex [49]. The binding of RNase H decreases the bending angle of the RDH duplex along its axis [50], which may contribute to its mechanisms for recognizing and cleaving ribonucleotides. The structural flexibility and bendability of an RDH duplex also provide insights into many intriguing findings, including the observation that the RNase H domain degrades the RNA strand of an RDH duplex more rapidly than that of an RNA duplex [51]. Previous studies revealed that structural changes in an RDH duplex affect the binding affinity of the RNA polymerase core enzyme [52]. Previous results also revealed that the bendability of an RDH duplex is affected by several factors, such as temperature [53], supercoiling [2], and adenine tracts [54]. Despite the biophysical importance of an RDH duplex and numerous studies on its mechanical properties, more evidence of direct measurements is needed to quantify its inherent structural flexibility and bendability.

In this study, we will measure the flexibility of an RDH duplex utilizing single-molecule cyclization experiments in comparison with that of the DNA duplex in the same sequences. The microscopic mechanisms underlying the difference in the flexibility between RDH and DNA duplexes will be revealed by analyzing the results from the all-atom molecular dynamics (MD) simulations. In addition, we will also perform single-molecule cyclization experiments using four RDH duplexes with sequences from two positive GC skew regions within the CGI promoter of the *APOE* gene, which is strongly associated with sporadic Alzheimer’s disease. This study aims to provide a deeper understanding of the structure and mechanical properties of an RDH duplex, as well as new insights into the formation of R-loop structures.

## 2. Materials and Methods

### 2.1. DNA and RDH Preparations

All oligonucleotides required to make the dsDNA and RDH duplexes were purchased from General Biosystem (Anhui) Co., Ltd (Anhui, China). The sequences of the oligonucleotides are listed in Section 2.4, including the DNA and RNA sequences used in smFRET experiments. The dsDNA and RDH duplexes in smFRET experiments were produced by mixing Cy5 and biotin-labeled DNA with Cy3-labeled complementary DNA or RNA at a molar ratio of 1:1.2 in an annealing buffer composed of 10 mM Tris–HCl (pH 8.0) and 1 mM EDTA. The mixtures were incubated at 95 °C for 5 min and then slowly cooled down to room temperature within 3 h. All annealed substrates were stored at −80 °C before use.

### 2.2. smFRET Data Acquisition

All smFRET experiments were carried out using a home-built, objective-type total internal reflection microscopy system (Nikon Corporation, Tokyo, Japan). A solid-state 532 nm laser (Coherent, Inc., Santa Clara, California, USA) and a 640 nm laser (Coherent, Inc., Santa Clara, California, USA) were used for smFRET measurements. The emission signal was separated by a dichroic mirror (cut-off = 630 nm) and was detected using an EMCCD camera (Andor Technology Ltd., Belfast, Northern Ireland). The coverslips (Fisher Scientific, Pittsburgh, PA, USA) and slides were progressively cleaned with Piranha solution, acetone, and sodium ethoxide. Then, the glass surfaces were coated with a mixture of 99% monomethoxy-polyethylene glycol (mPEG-2K-SVA, Huateng Pharma, Changsha, China) and 1% biotin-PEG (biotin-PEG2K-SVA, Huateng Pharma, Changsha, China).

Each flow cell was assembled using a larger slide, a smaller functional slide, and double-sided tape. A syringe equipped with a 2 mm inner diameter needle was utilized to apply room-temperature vulcanized silicone rubber (KE45T, Shinetsu, Tokyo, Japan) to both the inlet and outlet of the flow cell. After the rubber dried, Neutravidin (Thermo Scientific, Pittsburgh, PA, USA) (0.2 mg/mL) in 50 mM Tris-HCl (pH 8.0) was added to the flow cell and incubated for 10 min, followed by incubation with the biotinylated DNA or RDH duplexes for 10 min. In smFRET experiments, the surface density of DNA or RDH duplexes was carefully controlled to be sufficiently low to minimize the probability of dimer formation. Specifically, the immobilization concentration of biotinylated DNA or RDH duplexes was kept in the range of 50–100 pM, which corresponds to an estimated density of ~0.05–0.1 molecules/μm^2^. At this low surface density, we did not observe any FRET signals consistent with intermolecular interactions or dimerization.

Then, free DNA or RDH molecules were flushed out with an imaging buffer, which contained 50 mM Tris-HCl, 3 mM Trolox (Sigma), and an oxygen scavenging system (0.8% *w/v* D-glucose [Sigma], 0.1 mg/mL glucose oxidase [248,073 units/g; Sigma], as well as 0.02 mg/mL of catalase [2000–5000 units/mg; Sigma]) without cationic ions. Under this buffer condition, surface-immobilized DNA or RDH duplexes remained in an open conformation with no detectable energy transfer within our time resolution. The experiment commenced with the introduction of an imaging buffer identical in composition but supplemented with a high cation concentration (1 M NaCl). High concentrations of Na^+^ ions reduce the persistence length of both DNA and RDH duplexes, rendering them more flexible and thereby facilitating loop formation [42]. Additionally, high concentrations of Na^+^ ions reduce electrostatic repulsion between the negatively charged phosphate backbones of nucleic acid strands, thereby stabilizing the looped state by facilitating strand proximity and the hybridization of the 10 nt overhangs [55].

To prevent buffer acidification caused by the oxygen scavenging system, fresh imaging buffer was placed in the flow cell every 20 min. At each time point, approximately 2000–3000 molecules from different areas (50 µm × 25 µm) across the flow cell were analyzed using an imaging time resolution of 100 ms. All smFRET movies were collected using CellVision software (V1.4.0, Beijing Coolight Technology).

### 2.3. smFRET Data Analyses

The FRET efficiency was determined using the formula *I_A_*/(*I_D_* + *I_A_*), where *I_D_* and *I_A_* denote the fluorescence intensity of the donor and acceptor, respectively. At each time point, the FRET efficiency histograms from all molecules were fitted with two Gaussian distributions, and the proportion of looped population was defined as *A_H_*/(*A_H_* + *A_L_*), where *A_L_* and *A_H_* are the area under the fitted Gaussian curve at low FRET and high FRET, respectively. The fraction of looped molecules over time follows an exponential decay curve of the form $f\left(t\right)=A\left({1-e}^{-R\cdot t}\right)$. Here, *A* is the proportion of looped molecules at equilibrium, and *R* is the looping rate.

### 2.4. DNA and RNA Sequences Used in smFRET Experiments (5′-3′)

Bold T denotes the position of the biotin, attached internally to a thymine base. The underlined sequence denotes the sequence of the 10-nt overhang.

The dsDNA used in this study is as follows:

Cy5-acggattctgtgaaacggtggaggtgagga**T**agtcatgggtcaattagaagtcataggagaagtattaag

ggagtctatattgaggtactagc

Cy3-cagaatccgtgctagtacctcaatatagactcccttaatacttctcctatgacttctaattgacccatga

ctatcctcacctccaccgtttca

The RDH duplex used in this study is as follows:

Cy5-acggattctgtgaaacggtggaggtgagga**T**agtcatgggtcaattagaagtcataggagaagtattaag

ggagtctatattgaggtactagc

Cy3-cagaauccgugcuaguaccucaauauagacucccuuaauacuucuccuaugacuucuaauugacc

caugacuauccucaccuccaccguuuca

The C-DNA/G-RNA RDH-53 is as follows:

Cy5-cctccagtggctccaccttcacctgtggc**T**gagactcaactgtcaccccctcctctggctccatcccttc

cgtccccttttgcctctttctct

Cy3-ccacuggaggagagaaagaggcaaaaggggacggaagggauggagccagaggagggggugacagu

ugagucucagccacaggugaagguggag

The G-DNA/C-RNA RDH-53 is as follows:

Cy5-agagaaagaggcaaaaggggacggaaggga**T**ggagccagaggagggggtgacagttgagtctcagccaca

ggtgaaggtggagccactggagg

Cy3-cucuuucucuccuccaguggcuccaccuucaccuguggcugagacucaacugucacccccuccuc

uggcuccaucccuuccguccccuuuugc

The C-DNA/G-RNA RDH-49 is as follows:

Cy5-tccagaggcttcatctccggctccactggc**T**ccatcgcctccgtccctggctccatcattgccatctgtc

ccttttcttttttcctcttcttc

Cy3-agccucuggagaagaagaggaaaaaagaaaagggacagauggcaaugauggagccagggacggag

gcgauggagccaguggagccggagauga

The G-DNA/C-RNA RDH-49 is as follows:

Cy5-gaagaagaggaaaaaagaaaagggacaga**T**ggcaatgatggagccagggacggaggcgatggagccagtg

gagccggagatgaagcctctgga

Cy3-ccucuucuucuccagaggcuucaucuccggcuccacuggcuccaucgccuccgucccuggcucca

ucauugccaucugucccuuuucuuuuuu

### 2.5. All-Atom Molecular Dynamics Simulations

The simulations started with a DNA or RDH duplex in the same sequence of smFRET experiments. The initial straight structures of DNA and RDH were built using 3DNA [56] and the USFC Chimera package [57], respectively. The circularized DNA and RDH minicircles were then constructed by looping the straight structures using in-house scripts. Both DNA and RDH loops started as planar minicircles with the twist uniformly distributed between each base pair step, corresponding to linking number 9. The initial structure was solvated in a rectangle box filled with TIP3P water molecules. The charges of DNA/RNA were then neutralized with an appropriate number of Na^+^, and enough Na^+^ and Cl^−^ ion pairs were added to give a bulk salt concentration of 1 M.

All MD simulations were performed using the GROMACS 2021 software [58]. The OL21 and OL3 force fields were used for DNA [59] and RNA [60], respectively. The ion parameters from the Joung–Cheatham model were employed for monovalent ions [61]. Energy minimization was first applied to the simulation system starting with the steepest descent algorithm, which was followed by equilibration in the canonical (NVT) ensemble for 100 ps and in the isobaric–isothermal (NPT) ensemble for 10 ns. In equilibration, all heavy atoms were constraints with a harmonic potential of 1000 kJ/(mol∙nm^2^) to allow for the equilibration of ions around nucleic acids. After equilibration, the constraints on heavy atoms were removed, and simulations were carried out for 300 ns with a 2 fs time step. The conformations were saved every 10 ps, giving 30,000 snapshots in total. The last 200 ns of the production run was used for data analysis.

Periodic boundary conditions were applied in all three dimensions. A 1.0 nm cut-off was applied for van der Waals interactions and short-range electrostatic interactions. Long-range electrostatic interactions were handled via the particle mesh Ewald method [62]. All bonds containing hydrogen atoms were restrained using LINCS [63]. The temperature of the simulation system was coupled to 295 K using a V-rescale thermostat with a relaxation time of τ=0.1 ps, and the pressure was kept at 1 atm using Parrinello–Rahman pressure coupling with a relaxation time of τ=2.0 ps and compressibility of $4.5\times 10^{-5}$ bar^−1^.

## 3. Results

### 3.1. The Experimental Design of Single-Molecule RDH Duplex Cyclization

**Figure 1:**
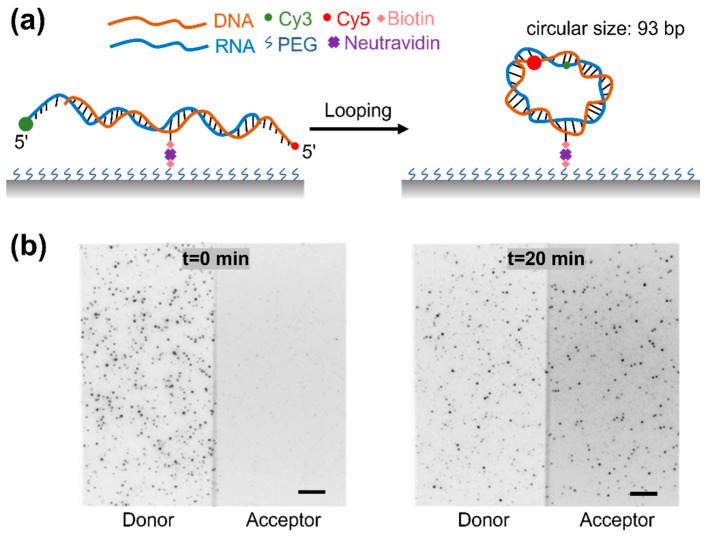
Experimental design. (**a**) Cy3- and Cy5-labeled RDH duplexes were anchored to glass surface via biotin–neutravidin interaction. (**b**) Fluorescence images of RDH duplexes in respective Cy3 and Cy5 channels are shown before (left panels) and 20 min after adding high salt (1 M NaCl) buffer (right panels). Scale bar, 5 µm.

We performed RDH cyclization experiments using single-molecule fluorescence resonance energy transfer (smFRET) for directly monitoring the cyclization of a single RDH duplex (Figure 1a) [64,65]. To prevent dimerization during prolonged observation, the RDH duplexes were anchored to the polymer-coated glass surface via a biotin linker positioned at an internal site of the DNA strand. Here, we used an RDH duplex with a circular size of 93 bp (the circular size refers to the total circumference of the RDH duplex circle formed after cyclization, comprising both the initial duplex length and the length of the overhang). An RNA strand labeled with Cy3 (donor) at the 5′ end and a DNA strand labeled with Cy5 (acceptor) at the 5′ end were annealed to form an RDH duplex with a 10 nt overhang at both 5′ ends. The single-stranded overhangs of the RDH duplex are complementary so that the hybridization will trap the RDH duplex in the looped state. In the unlooped state, the donor and acceptor are distant from each other, and the RDH duplex exhibits no FRET signal. The cyclization results in the spatial proximity of the fluorescent dyes show that the RDH duplex exhibits a high FRET signal. Thus, the FRET value and the relative intensities of Cy3 and Cy5 allow for a clear distinction between the unlooped and looped states (Figure 1b).

### 3.2. The High Bendability of Short RDH Duplex

**Figure 2:**
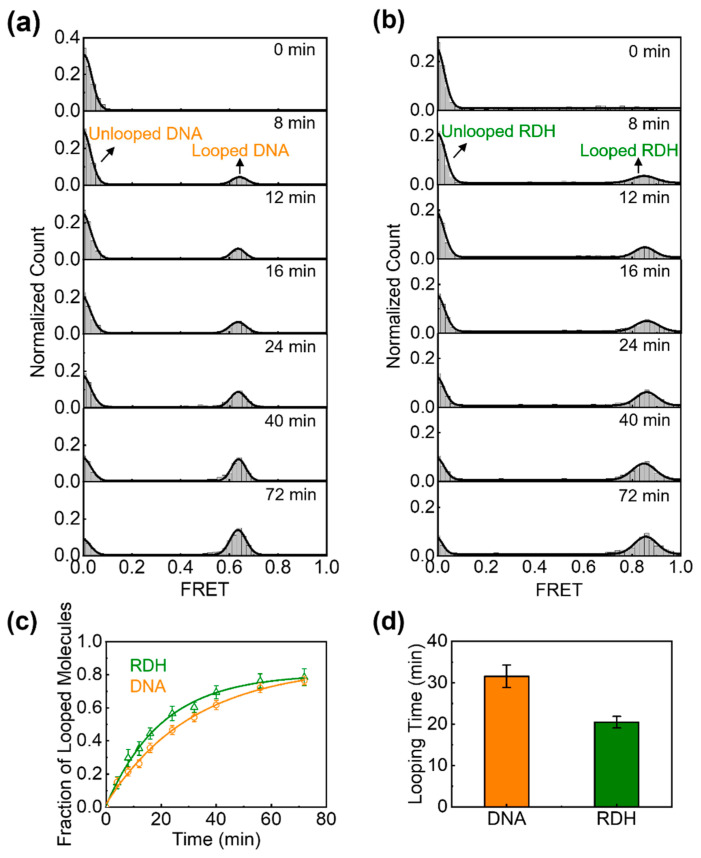
Quantitative characterization of the flexibility of DNA (orange) and RDH (olive) duplexes with the same sequences. (**a**,**b**) Histograms of FRET efficiency as a function of time (with t = 0 marking the addition of high-salt buffer) show the temporal progression of looped (high FRET) and unlooped (low FRET) populations for (**a**) DNA and (**b**) RDH duplexes. (**c**) The fraction of looped DNA or RDH duplexes (high FRET population) as a function of time, measured from the histograms in (**a**,**b**). An exponential fit to each curve gives the looping rate *R*. (**d**) The measured looping time (1/*R*) of the DNA and RDH duplexes. The error bars represent the SE from more than three experiments.

To quantify the flexibility of DNA and RDH duplexes, we recorded the fraction of unlooped and looped molecules in real time following methods used in previous studies [65,66,67]. The single-molecule cyclization experiment was initiated in an ion-free buffer in order to strongly favor the unlooped state. The subsequent addition of a buffer containing high concentrations of Na^+^ ions can facilitate the stabilization of the looped state. Clearly, the proportion of high FRET populations corresponding to the looped molecules increases over time for both DNA and RDH duplexes (DNA in Figure 2a, RDH in Figure 2b). The looping process was nearly irreversible, with the high FRET state progressively accumulating and reaching saturation in approximately one hour (Figure 2c). In this case, the looping rate *R* could be determined by fitting the temporal progression of the looped population with a single exponential function $f\left(t\right)=A\left({1-e}^{-R\cdot t}\right)$. Here, *A* is the proportion of looped molecules at equilibrium, and *R* is the looping rate used as a measure of DNA or RDH duplex flexibility. An increased looping rate indicates greater structural flexibility of the duplex. We calculated the looping time (1/*R*) of the DNA and RDH duplexes and found that the looping time of the DNA duplex is approximately 1.6-fold higher than that of the RDH duplex (Figure 2d). These results indicate that the RDH duplex is more flexible than the DNA duplex with the same sequence on a short length scale (circle size: 93 bp).

### 3.3. The Curvature and Structural Fluctuations of an RDH Duplex

**Figure 3:**
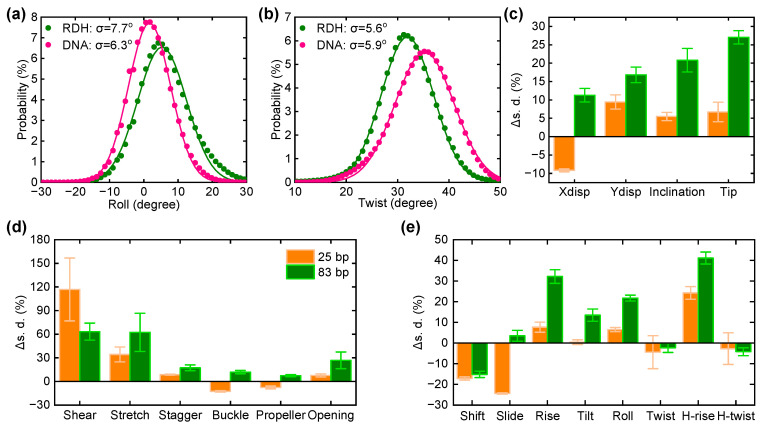
Molecular dynamics simulations of straight 83 bp RDH and DNA. (**a**) The distributions of the roll angle for 93 bp RDH and DNA. The solid lines are Gaussian fit to the simulation data. Standard deviations are shown in the legend. The average values of the roll angle are 6.0° and 1.9° for RDH and DNA, respectively. (**b**) The same as (**a**) but for the twist angle. The average values of the roll angle are 31.9° and 34.8° for RDH and DNA, respectively. (**c**–**e**) The average change in the standard deviations of axis, intra-base pair, and inter-base-pair parameters of RDH relative to the corresponding values of DNA. The data of 25 bp is shown in orange, and the data of 83 bp is shown in olive. The error bars are obtained from the average with 10 qual intervals after equilibrium.

To understand the higher flexibility of RDH compared to DNA, we performed all-atom MD simulations of the central 83 bp and 25 bp fragments from the experimental construct for both RDH and DNA in a 1 M NaCl solution at room temperature. We choose the length of 83 bp to exclude the effect of overhang, and the 25 bp duplex was used to identify the structural properties of nucleic acid duplex with the length scale studied in most simulations. Each simulation was run for 300 ns, and we collected the trajectory of last 200 ns for data analysis. The configuration of RDH or DNA was sampled every 10 ps, giving 20,000 ensembles of coordinates in total. We calculated and recorded the geometric parameters using Curves+, V3.0 [68], including the base pair axis parameters (Xdisp, Ydisp, inclination, and tip), intra-base pair parameters (shear, stretch, stagger, buckle, propeller, and opening), and inter-base pair parameters (roll, tilt, twist, slide, shift, and rise). The flexibility of RDH and DNA can be analyzed using these structural parameters because the distribution of microscopic excursions could cause big differences in macroscopic elastic parameters. Among these parameters, roll and twist is strongly correlated with the bending of the duplex [65,69]. Thus, we first analyzed their distributions and calculated the average value of roll and twist. As shown in Figure 3a, the roll angle increased from 1.9° in 83 bp DNA to 6.0° in 83 bp RDH, indicating an obvious change in the intrinsic curvature for RDH. In addition, we found that twist decreases from 34.8° in 83 bp DNA to 31.9° in 83 bp RDH (Figure 3b). Unwinding the duplex ubiquitously facilitates the bending of RDH. The roll and twist angles are also increased in 25 bp RDH compared to 25 bp DNA (Figure S1), suggesting the weak length dependence of the intrinsic geometry.

Due to thermal fluctuations, the distribution of the structural parameter is Gaussian, while the extracted standard deviations describe the flexibility. Normally, the broadening of a Gaussian distribution gives a larger standard deviation, which manifests the increased looping kinetic with the larger fluctuation around its average structure [66]. We calculated the standard deviations from the Gaussian distributions for all structural parameters, such as the distributions of roll and twist in Figure 3a,b. As shown in Figure 3c–e, the standard deviations of most structural parameters (except shift, twist, and H-twist) are increased in 83 bp RDH compared to those of DNA. As a result, the structural fluctuations in RDH are significantly higher than those in DNA, leading to the increased flexibility of the RDH duplex. As a control, we also analyzed the structural fluctuations in 25 bp RDH and DNA. The standard deviations of three axis parameters (Ydisp, inclination, and tip), two intra-base pair parameters (shear and stretch), and one inter-base pair parameter (H-rise) are mainly increased in the 25 bp RDH relative to the values in the 25 bp DNA. The increased structural fluctuations also contribute to the looping kinetics in RDH. Other structural parameters are barely changed, while Xdisp, shift, and slide were found to be decreased in the 25 bp RDH, indicating that the circular length could also affect the dynamics of RDH and DNA fragments.

### 3.4. Kink Release Bending Stress in RDH Duplex

**Figure 4:**
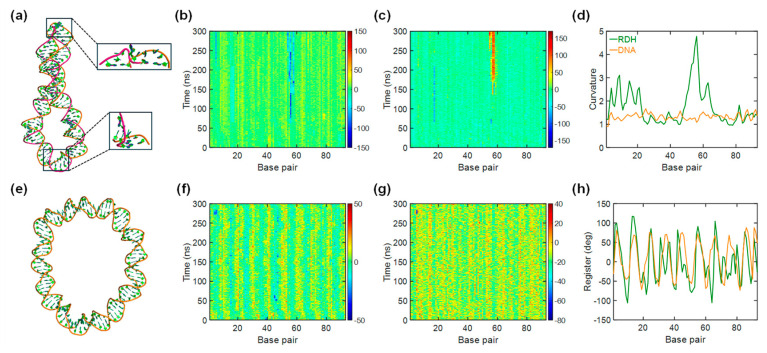
Molecular dynamics simulations of 93 bp RDH and DNA minicircles. (**a**) The final snapshot of the molecular structure of RDH. The DNA strand is shown in orange, and the RNA strand is shown in magenta. The upper box shows the formation of type II kinks with 54–58 base pair steps. The lower box shows the formation of type I kinks with 5–6 base pair steps. (**b**) The time evolution of the roll angle for RDH. Base-pair steps are numbered from left to right along the horizontal axis. (**c**) The time evolution of the propeller angle for RDH. Base pairs are numbered from left to right along the horizontal axis. (**d**) The average curvature with the minicircle base pair. (**e**) The final snapshot for the molecular structure of DNA. (**f**) The same as in (**b**) but for DNA. (**g**) The same as in (**c)** but for DNA. (**h**) The average register with the minicircle base pair.

We also performed MD simulations to show the structural dynamics of the 93 bp RDH and DNA minicircle, with both initial structures remaining planar and closed (LK = 9). Initially, the twist angle is uniformly distributed in each base pair step. Figure 4a shows the final structures of the RDH minicircle after a 300 ns production run, where kinks can be visually identified within regions of high curvature (highlighted in black boxes of Figure 4a). In contrast, the DNA minicircle remains almost unkinked during the whole simulation time. There are also no visually obvious structural disruptions in the DNA loop, except it was weakly deformed into an elliptical shape (Figure 4e). Our result regarding the DNA minicircle agrees with previous simulations [70,71] and experiments [72,73] showing that kinked structures are not typically found in torsional relaxed DNA. The rare events of DNA openings are energetically disfavored and less possible to observe for a production run of 300 ns.

To characterize the formation of kinks in detail, we analyzed the structural parameters of both RDH and DNA. Figure 4b,c,f–g present the time series of the roll and propeller angle for RDH and DNA loops. For the DNA loop, the roll and propeller values of all base pair steps remain within the standard ranges in all simulation times (Figure 4f,g). For the RDH loop, the 54–58 base pair steps adopt a large negative roll and large positive propeller angles (Figure 4b,c), which are in accordance with the formation of type II kinks in previous studies [70,74]. A detailed time evolution of roll and propeller angles at these base pair steps can be found in Figure S2. The RDH loop also shows a kink with base pair step 5, which is similar to the type I kink form. The average time values of the structural parameters for the 93 base pair steps also manifest the formation of kink structures at these base pair steps (Figure S3).

The formations of kinks can be an effective way to save energy costs to strongly bend RDH. Considering the fewer probability of kinks in DNA minicircles, the strongly bent DNA would be energetically unfavorable compared to RDH. We analyzed the curvature and register in RDH and DNA minicircles (Figure 4d,h). In general, kinks tend to appear in regions exhibiting high curvature. By introducing this high curvature obtained from breaking base pairs, the kinks distort the circularized loops. The register defines the local curvature direction with respect to a given base pair, enabling us to identify the deformation direction towards a major groove or minor groove. A register value of −90° corresponds to bending into a major groove, and a value of 90° corresponds to bending into a minor groove. The larger absolute register values of RDH are placed inward in the center of the minicircle. We also carried out MD simulations of DNA minicircles with LK = 10, whose conformation evolved toward the characteristic change in the roll and propeller when kinks formed at the 30 base pair step (Figure S4).

### 3.5. The Sequence Dependence in the Bendability of the RDH Duplex

**Figure 5:**
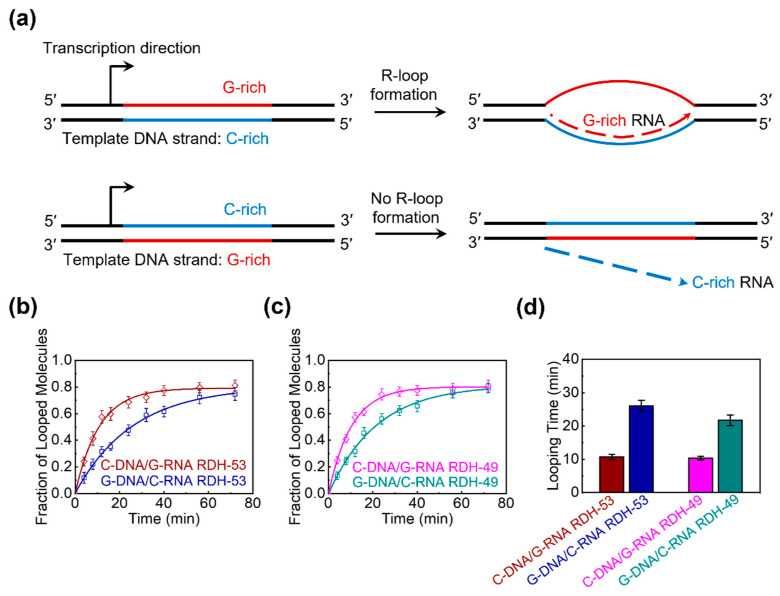
The RDH duplex composed of C-rich DNA and G-rich RNA shows higher flexibility than that composed of G-rich DNA and C-rich RNA. (**a**) Transcription through regions of GC skew such that G-rich RNA is generated can lead to R-loop formation (top). In contrast, transcription through the same region such that C-rich RNA is produced does not lead to R-loop formation (bottom). The G-rich and C-rich strands are color-coded in red and blue, respectively. The RNA strand is represented by a dashed line, with the arrow indicating the direction of RNA generation. (**b**,**c**) The fraction of looped RDH duplexes as a function of time. C-DNA/G-RNA RDH refers to RDH composed of C-rich DNA and G-rich RNA, while G-DNA/C-RNA RDH refers to RDH composed of G-rich DNA and C-rich RNA. Two positive GC skew regions (93 bp each) within the CGI promoter of *APOE* gene were analyzed, with GC skew values of 0.53 (**b**) and 0.49 (**c**), respectively. An exponential fit to each curve gives the looping rate *R*. (**d**) The measured looping time (1/*R*) of all four RDH duplexes shown in (**b**,**c**). The error bars represent the SE from more than three experiments.

It has been reported that transcription across GC-skewed regions leads to the formation of R-loops, which protects CGI promoters from DNA methylation [6]. Moreover, R-loop structures form in *cis* when the nascent G-rich RNA transcript rehybridizes with the complementary C-rich template DNA strand, displacing the non-template G-rich DNA strand into a predominantly single-stranded state (Figure 5a). To determine whether the flexibility of the RDH duplex formed by the binding of G-rich RNA to the C-rich DNA template provides an advantage in R-loop formation, we performed single-molecule cyclization experiments using four RDH duplexes. Specifically, we chose two positive GC skew regions (93 bp each) within the CGI promoter of the *APOE* gene, which is strongly associated with sporadic Alzheimer’s disease [6,75]. The GC skew values for the two regions are 0.53 and 0.49, respectively. In the region with a GC skew value of 0.53, each DNA strand hybridizes with its complementary RNA strand, resulting in two RDH duplexes: C-rich DNA/G-rich RNA RDH-53 and G-rich DNA/C-rich RNA RDH-53 (Figure 5b). Similarly, in the region with a GC skew value of 0.49, two analogous RDH duplexes, C-rich DNA/G-rich RNA RDH-49 and G-rich DNA/C-rich RNA RDH-49, were obtained (Figure 5c). The circular size of each RDH duplex after looping is 93 bp, consisting of an 83 bp initial RDH duplex length and a 10 bp overhang length. The looping rate *R* for each RDH duplex in Figure 5b,c was obtained using the same experimental measurement and data fitting approach as that used in Figure 2b,c. Then, we calculated the looping time (1/*R*) of all four RDH duplexes and found that the looping time of G-rich DNA/C-rich RNA RDH-53 is approximately 2.4-fold higher than that of C-rich DNA/G-rich RNA RDH-53, while the looping time of G-rich DNA/C-rich RNA RDH-49 is approximately 2.1-fold higher than that of C-rich DNA/G-rich RNA RDH-49 (Figure 5d). Together, these results indicate that the RDH duplex composed of C-rich DNA and G-rich RNA is more flexible than that composed of G-rich DNA and C-rich RNA in the same CGI promoter region, suggesting that the superior flexibility of the RDH duplex composed of C-rich DNA and G-rich RNA may contribute to R-loop formation in the regions of GC skew, thereby protecting CGI promoter sequences from DNA methylation.

## 4. Discussion

In this work, we quantitatively measured the bending flexibility of a RDH duplex using smFRET and compared it with that of a DNA duplex with the same sequence. Our single-molecule cyclization experiments revealed that at a high concentration of cations (1 M NaCl), the RDH duplex is more flexible than the DNA duplex on the short length scale (circular size: 93 bp) (Figure 2). The increased flexibility of the RDH duplex over the DNA duplex is consistent with previous fluorescence measurements [76] and molecular dynamics simulations [44,77].

Notably, this finding seems to contradict the generally accepted view that the RDH duplex is shorter and more rigid than the DNA duplex due to the larger persistence length, smaller stretch modulus, and shorter contour length of RDH [42]. Our all-atom MD simulations provided new insights into the mechanistic underpinnings of this phenomenon. We revealed that the increased bendability of RDH is related to both the increased intrinsic curvature and structural fluctuations (Figure 3). The larger structural fluctuations of RDH suggest that it enhances flexibility by allowing for easier local deformations, particularly at the helical junctions, which contribute to an overall increase in the bendability of the RDH duplex. In addition, our simulations of circular loops revealed typical kinks in the RDH minicircle, which contrasted the DNA minicircle that remained unkinked during the simulation time (Figure 4). The structural disruption of RDH promotes easier bending compared to the DNA duplex. The structural parameters confirm the occurrence of kinks, suggesting possible routes to release bending stress in the RDH duplex. This structural flexibility may be due to the differences in sugar pucker conformations between the RNA strand and DNA strand. The C3′-endo sugar pucker in the RNA strand and the C2′-endo sugar pucker in the DNA strand create an inherent asymmetry, facilitating easier bending by allowing the RDH duplex to accommodate different conformational states.

The bendability and structural flexibility of the RDH duplex could be significant in the biological processes involving the RDH duplex, such as R-loop formation [8,9]. During transcription, a nascent ssRNA anneals to its template DNA, giving rise to the formation of an R-loop, which consisted of an RDH and a displaced ssDNA. The GC-skewed regions particularly favor the formation of R-loops [6,7], while regions lacking GC skew during transcription do not demonstrate R-loop formation [6]. Previous studies revealed that the promotion behind R-loop formation is the superior thermodynamic stability of RDH between G-rich RNA and C-rich DNA templates [38]. In the R-loop structure, the displaced ssDNA often contains a G-rich sequence and forms a secondary structure. Thus, the extended RDH duplex is generally bent by the ssDNA; thus, the high bendability of the RDH duplex could contribute to the formation of an R-loop [8,9]. In this work, we found that the RDH duplex composed of a C-rich DNA strand and a G-rich RNA strand showed notably higher bendability than that composed of a G-rich DNA strand and a C-rich RNA strand in the same CGI promoter regions (Figure 5), suggesting that a possible route to protect CGI promoter sequences from DNA methylation is through the high flexibility of a RDH duplex composed of a C-rich DNA strand and a G-rich RNA strand. Guanine (G) has two aromatic rings, while pyrimidine cytosine (C) has only one aromatic ring, making the base stacking interactions stronger in G-rich strands but weaker in C-rich strands (Figure S5). Thus, a G-rich DNA strand is more rigid and tends to retain its B-form conformation, which conflicts the A-form RNA strand and reduces the overall flexibility of RDH. The C-rich DNA strand is less restricted by the base-stacking interactions, allowing for more freedom to vary the backbone in RDH. This is demonstrated by both the north and south conformations of sugar puckering in RDH composed of C-rich DNA and G-rich RNA [78,79]. As a result, RDH composed of C-rich DNA and G-rich RNA is more stable than that composed of G-rich DNA and C-rich RNA, but it possess higher flexibility due to the larger conformational dynamics of a C-rich DNA strand. These sequence-dependent variations in bendability suggest that the local sequence context of the RDH duplex can play a critical role in modulating the mechanical properties of RDH duplexes. In this study, we selected two positive GC skew regions within the CGI promoter of the *APOE* gene, aiming to provide an initial proof of concept for the sequence-dependent bendability of RDH duplexes. Nevertheless, further validation using a broader set of sequences from diverse genomic loci and with varying degrees of GC skew is planned in future studies to strengthen the generality of our conclusions and establish a more comprehensive understanding of the relationship between sequence composition, RDH flexibility, and R-loop formation. It will be intriguing to explore the specific mechanisms of this sequence-dependent effect in the bendability of these RDH duplexes.

This study holds potential for broad extension as duplex flexibility may be sensitive to salt conditions, the GC content, temperature, or modifications [37,38,39,40,80,81]. Furthermore, the flexibility of an RDH duplex plays a critical role in protein–nucleic acid recognition [32]. For instance, RNase H distinguishes an RDH duplex from an RNA duplex based on the different bendability patterns in the duplexes [36]. How do cellular proteins that interact with RDH duplexes, such as those involved in transcription or replication, recognize and exploit the bendability of RDH duplex? Future studies could investigate the interactions between RDH duplexes and various RDH-binding proteins, such as RNase H cleavage or CRISPR-Cas9 binding, which will be crucial for understanding the functional significance of RDH duplexes in biological processes.

Our findings will provide valuable insights into the comparative structural features and flexibility of RDH and DNA duplexes. This work provides novel biophysical insights into the mechanism of R-loop formation, a process critical for the biological functions of an RDH duplex. Targeting the inherent structural flexibility of an RDH duplex may provide new therapeutic avenues, such as small-molecule destabilizers to disrupt pathogenic R-loops involved in critical cellular processes, such as transcriptional and replication regulation.

## 5. Conclusions

In summary, we measured the flexibility of an RDH duplex using single-molecule cyclization experiments in comparison with that of a DNA duplex with the same sequences. Our single-molecule experiments showed that the RDH duplex is more flexible than the DNA duplex on the short length scale (circular size: 93 bp). Our molecular dynamics simulations demonstrated that the RDH duplex has larger intrinsic curvature and structural fluctuations and more easily forms kinks than DNA, which promote the bending flexibility of RDH from unlooped structures. In addition, we discovered that the RDH duplex composed of a C-rich DNA strand and a G-rich RNA strand is more flexible than that composed of a G-rich DNA strand and a C-rich RNA strand through single-molecule cyclization experiments. Our findings provide a deeper understanding of the structures and flexibility of RDH duplexes as well as novel biophysical insights into the mechanism of R-loop formation.

## Data Availability

The original contributions presented in this study are included in the article/Supplementary Materials. Further inquiries can be directed to the corresponding author.

## Supplementary Materials

The following supporting information can be downloaded at https://www.mdpi.com/article/10.3390/biom15050724/s1; Figure S1: The distributions of the roll angle and twist angle for 25 bp RDH and DNA; Figure S2: The time evolution of the roll angle and propeller angle at the 58 base pair step for the RDH minicircle; Figure S3: The average values of structural parameters as a function of the base pair step for RDH and DNA minicircles; Figure S4: Molecular dynamics simulations of 93 bp DNA minicircles with LK = 10; Figure S5: Sequence-dependent structural differences in 12 bp RDH duplexes and G-C base pairing.
