# Supplementary material for: High Bendability of Short RNA-DNA Hybrid Duplex Revealed by Single-Molecule Cyclization and Molecular Dynamics Simulations
Source: Biomolecules. 2025 May 15;15(5):724. doi: 10.3390/biom15050724 (PMC12109412; doi:10.3390/biom15050724)
Supplement: Supplementary file 1 [file biomolecules-15-00724-s001.zip › biomolecules-3624822-supplementary.pdf]

Supplemental Figures

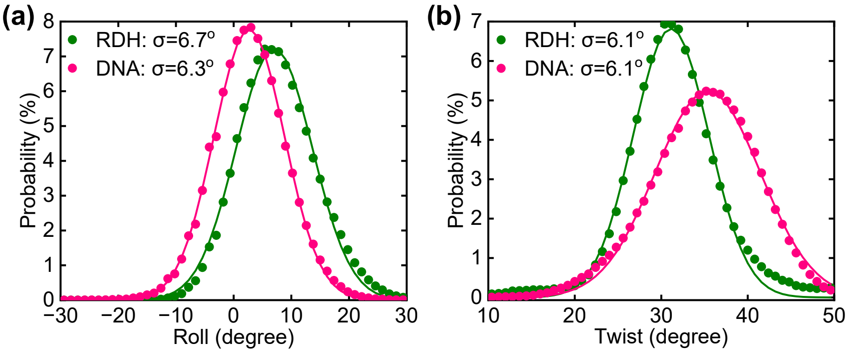

Figure S1. (**a**) The distributions of roll angle for 25-bp RDH and DNA. The solid lines are Gaussian fit to the simulation data. Standard deviations are shown in the legend. The average values of roll angle are 7.6° and 2.8° for RDH and DNA, respectively. (**b**) Same as in **a** but for the twist angle. The average values of roll angle are 31.6° and 34.8° for RDH and DNA, respectively.

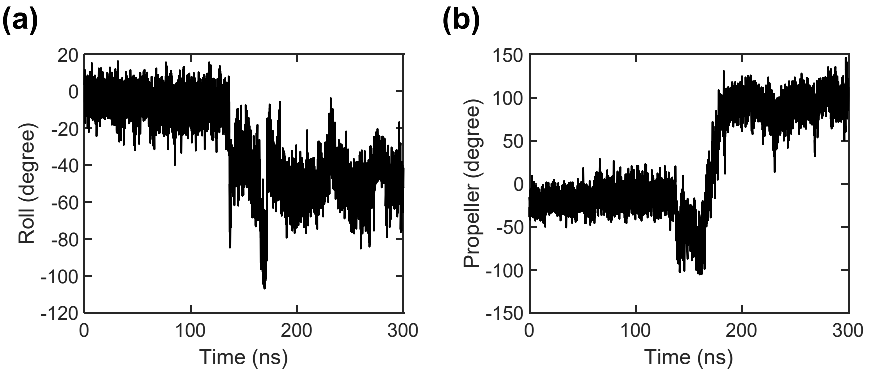

Figure S2. (**a**) Time evolution of roll angle at the 58 base pair step for RDH minicircle. (**b**) Same as in **a** but for the propeller angle.

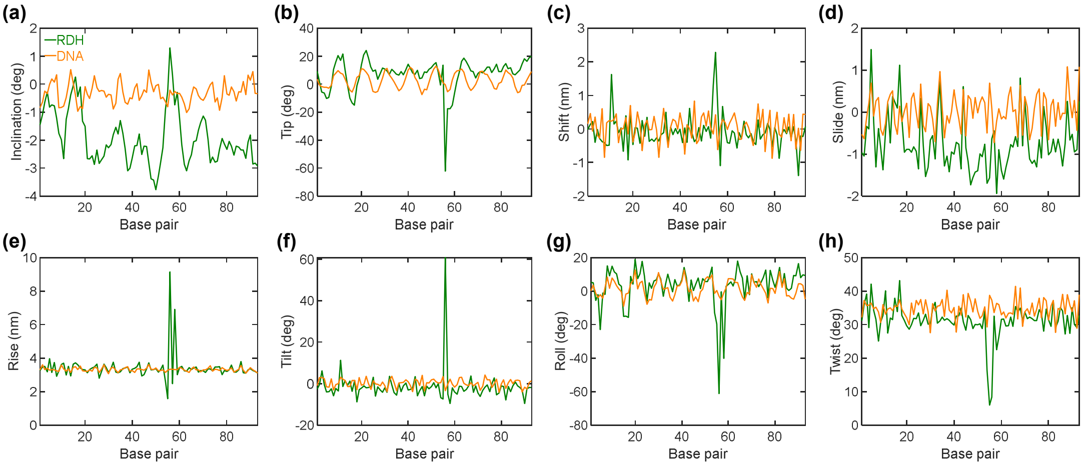

Figure S3. The average values of structural parameters as a function of base pair step for RDH and DNA minicircles: (**a**) Inclination, (**b**) Inclination, (**c**) Shift, (**d**) Slide, (**e**) Rise, (**f**) tilt, (**g**) Roll, and (**h**) Twist.

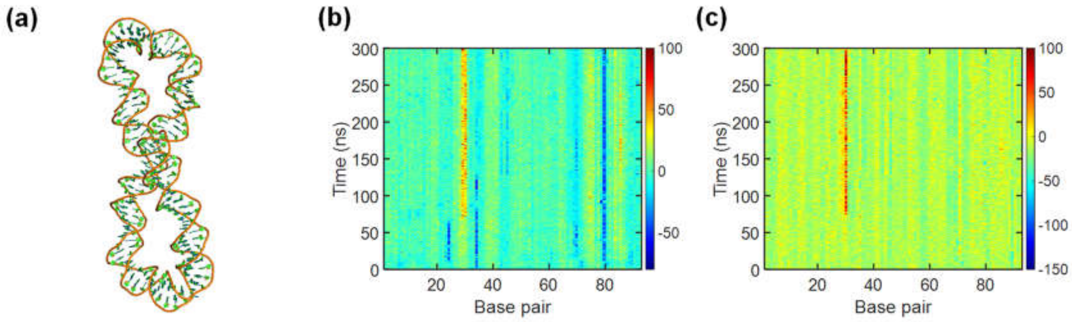

Figure S4. Molecular dynamics simulations of 93-bp DNA minicircles with LK=10. (**a**) Final snapshot for the molecular structure of DNA. (**b**) Time evolution of roll angle for DNA minicircle. (**c**) Time evolution of propeller angle for RDH. Base-pairs are numbered from left to right along the horizontal axis.

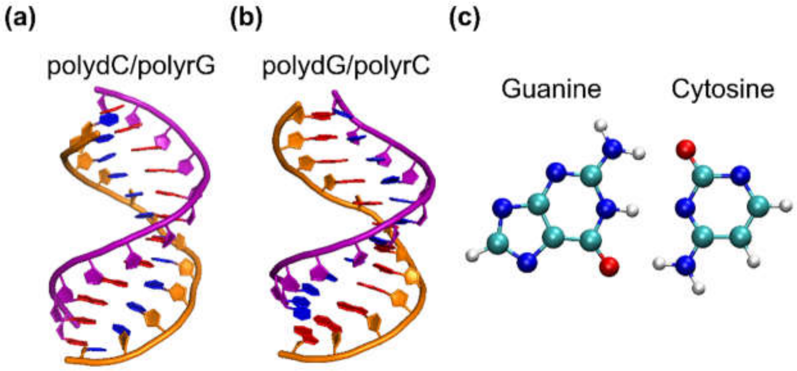

Figure S5. Comparison of the 12-bp RDH structures for (**a**) dC/rG and (**b**) dG/rC from simulations. (**c**) Illustration of the chemical structure for G-C base pair. The DNA backbone is shown in orange and the RNA backbone is shown in purple. The base G is shown in red and base C is shown in blue.
